# To Explore the Inhibitory Mechanism of Quercetin in Thyroid Papillary Carcinoma through Network Pharmacology and Experiments

**DOI:** 10.1155/2022/9541080

**Published:** 2022-12-03

**Authors:** Ying Sun, Wenjun Xie, Ning Kang, Jiaoyu Yi, Xianhui Ruan, Linfei Hu, Jingzhu Zhao, Xiangqian Zheng, Songfeng Wei, Ming Gao

**Affiliations:** ^1^Department of Thyroid and Neck Tumor, Tianjin Medical University Cancer Institute and Hospital, National Clinical Research Center for Cancer, Key Laboratory of Cancer Prevention and Therapy, Tianjin's Clinical Research Center for Cancer, Tianjin 300060, China; ^2^Department of Head and Neck Tumor, Chongqing University Cancer Hospital, Chongqing 400030, China; ^3^Department of General Surgery, Shengli Clinical Medical College, Fujian Provincial Hospital, Fuzhou 350001, China; ^4^Department of Breast and Thyroid Surgery, Tianjin Union Medical Center, Tianjin 300121, China; ^5^Tianjin Key Laboratory of General Surgery in Construction, Tianjin Union Medical Center, Tianjin 300121, China

## Abstract

Quercetin, a flavonoid with anti-inflammatory and anticancer properties, is expected to be an innovative anticancer therapeutic agent for papillary thyroid carcinoma (PTC). However, the downstream signaling pathways that mediate quercetin-dependent anticancer properties remain to be deciphered. Herein, potential targets of quercetin were screened with several bioinformatic avenues including PharmMapper, Gene Expression Omnibus (GEO) database, protein–protein interaction (PPI) network, and molecular docking. Besides, western blot, CCK-8 transwell analysis of migration and invasion, flow cytometric analysis, and colony formation assays were performed to investigate the underlying mechanism. We found four core nodes (MMP9, JUN, SPP1, and HMOX1) by constructing a PPI network with 23 common targets. Through functional enrichment analysis, we confirmed that the above four target genes are enriched in the TNF, PI3K-AKT, and NF-*κ*B signaling pathways, which are involved in the inflammatory microenvironment and inhibit the development and progression of tumors. Furthermore, molecular docking results demonstrated that quercetin shows strong binding efficiency with the proteins encoded by these 4 key proteins. Finally, quercetin displayed strong antitumor efficacy in PTC cell lines. In this research, we demonstrated the application of network pharmacology in evaluating the mechanisms of action and molecular targets of quercetin, which regulates a variety of proteins and signaling pathways in PTC. These data might explain the mechanism underlying the anticancer effects of quercetin in PTC.

## 1. Introduction

Thyroid cancer, the most common malignant tumor of the endocrine system, is one of the fastest growing tumors in the world [1] and is increasing 3.4% annually [2]. Papillary thyroid cancer (PTC) is the most common type of thyroid cancer, and most patients have a good prognosis after surgery, thyroid-stimulating hormone suppression, and/or radioactive iodine therapy [3]. However, 5% of patients finally get distant metastasis [4] which means worse prognosis. The reasons are still unclear, and earlier detection of PTC is urgently needed [5]. The main reason for the progression of these patients is probably the dedifferentiation of the tumor cells. Due to dedifferentiation of tumor cells, patients have reduced expression and abnormal function of thyroid-stimulating hormone receptor (TSHR) and natrium iodide symporter (NIS), resulting in insensitivity to 131I therapy and thus becoming iodine-refractory differentiated thyroid cancer with poor prognosis. Recent studies have suggested the relationship between the immune microenvironment and tumor aggressiveness [6].

Quercetin is a flavonoid that is found in various fruits and vegetables. It has antiviral, antioxidant, and antiallergic properties and a variety of pharmacological effects [7]. Moreover, quercetin is well known for its anticarcinogenic potential; the mechanism is involved in intercalating DNA in cancer cell lines [8] or binding to cellular receptors [9]. Previous studies have shown the cancer prevention and treatment function of quercetin in colon cancer [10] and cervical cancer [11].

Network pharmacology is a method for drug design that uncovers complex relationships between multiple components and targets, and it contains network analysis, systems biology, redundancy, connectivity, and pleiotropy [12]. Various studies have demonstrated the power of network analysis in understanding biological systems. Network pharmacology enables effective elucidating of previously unexplored natural products and provides systematic methods to extend the space of drug treatment in various diseases [13].

**Figure 1:**
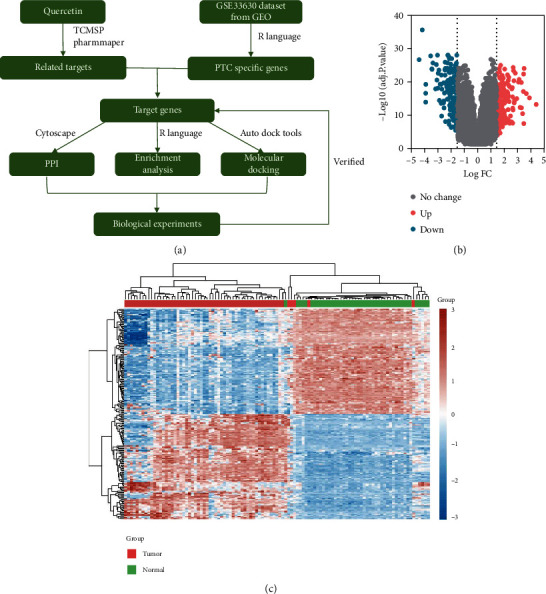
(a) Flowchart of the analyses in this study. PTC: papillary thyroid carcinoma; TCMSP: Traditional Chinese Medicine Systems Pharmacology; GEO: Gene Expression Omnibus; PPI: protein–protein interaction. (b) A volcano plot of DEGs. There were 423 significantly downregulated genes (blue dots) and 481 significantly upregulated genes (red dots) between the normal group and the tumor group (adjusted *p* value < 0.05 and |logFC| > 1.5). (c) A heat map of DEGs. DEGs in the two groups are indicated by green (downregulated genes) and red (upregulated genes). DEGs: differentially expressed genes.

As quercetin exerts multiple pharmacological effects and multiple targets, therefore, we try to find a method by which we can systematically elucidate the role of quercetin. Protein–protein interaction (PPI) applies to the process by which protein molecules constitute a protein complex by noncovalent bonds. The interactions between drugs and specific nodes or modules can be elucidated to identify potential mechanisms using PPI network [14]. Besides, we performed molecular docking for determining protein-ligand interactions. In the study, we sought to verify the role of quercetin on PTC and the associated signaling pathway through network pharmacology and then verified the proteins in the pathway to provide rationale for anti-PTC therapy. The roadmap of this study is shown in Figure 1(a).

## 2. Results

### 2.1. DEGs in PTC

105 samples were downloaded from the GEO database, which were categorized as two groups: the tumor group including 60 PTC samples and the normal group including 45 normal thyroid samples. Compared to the normal group, 904 DEGs were obtained in the cancer group, containing 481 downregulated genes and 423 upregulated ones. The volcano plot showed the DEGs identified between normal group and tumor group samples (Figure 1(b)). The heat map showed the expression of the DEGs (Figure 1(c)). The 904 DEGs were cross-fertilized with the quercetin targets analyzed by the database for subsequent construction of the protein network.

### 2.2. Potential Targets of Quercetin in PTC

**Figure 2:**
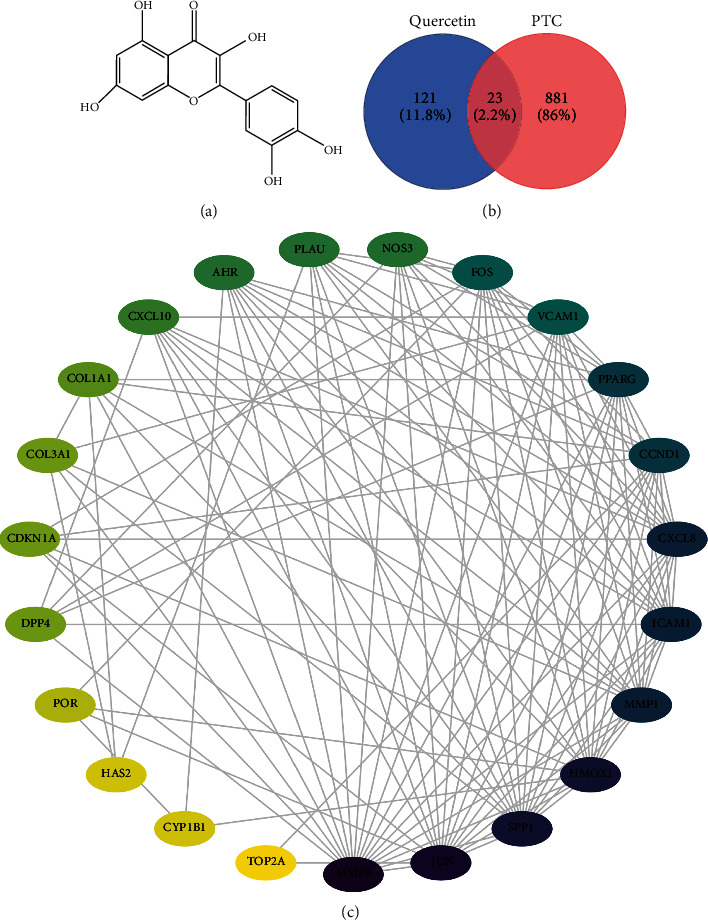
Potential targets of quercetin against PTC. (a) 2D molecular structure of quercetin. (b) Venn diagram summarizing the common targets correlated with quercetin and PTC. PTC: papillary thyroid carcinoma. (c) The PPI of candidate targets against PTC. A protein–protein interaction (PPI) network of quercetin target proteins. The PPI network contained 23 nodes and 121 edges (average node degree was 9.68, and the local clustering coefficient was 0.67 in the PPI network).

144 targets of quercetin were obtained by utilizing the online database, and 23 common targets, which are relevant to both quercetin and PTC, were filtered out by Venn diagram (Figure 2(b)). The 23 targets were used for the establishment of PPI network.

To clarify the connections among the 23 target proteins, we constructed a PPI network (Figure 2(c)) to show the correlation of the tumorigenesis and development of PTC. The PPI network had 23 nodes and 121 edges, with a local clustering coefficient of 0.67 and an average node degree of 9.68. The color reflected the degree of gene contribution, indicating whether there is a positive correlation with the occurrence of PTC. The nodes of MMP9, JUN, SPP1, HMOX1, and MMP1 were significantly enriched. The top 10 genes highly expressed in the PPI network were sorted by degree, and the specific data are shown in Table 1. The top 4 genes (MMP9, JUN, SPP1, and HMOX1) were selected based on the results for further experiments.

### 2.3. KEGG Pathway and GO Functional Enrichment Analyses

**Figure 3:**
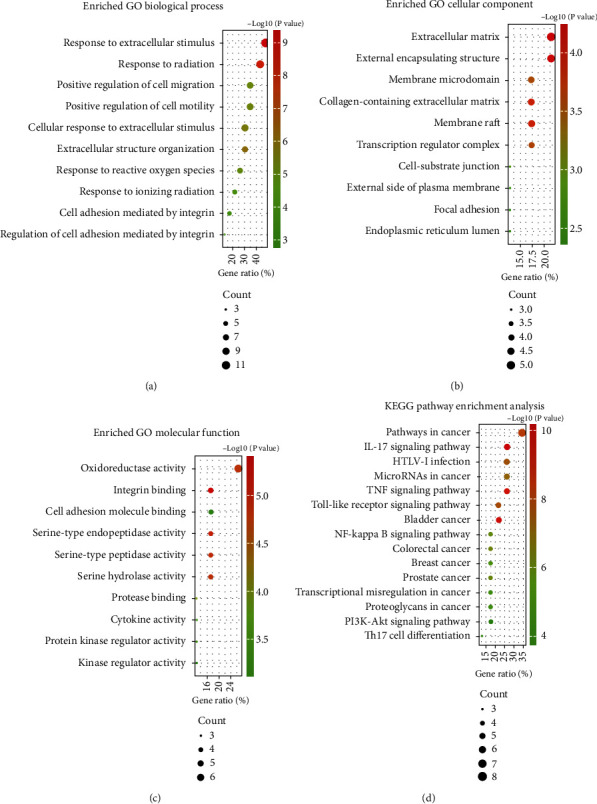
GO functional and KEGG pathway enrichment analyses. (a-c) GO enrichment analysis including biological process (BP), molecular function (MF), and cellular component (CC) of 23 specialized targets; (d) KEGG pathway enrichment analysis of 23 specialized targets.

The 23 commonly regulated genes were further analyzed to ascertain their biological processes. Biological processes with high enrichment scores included response to extracellular stimuli, oxidoreductase activity, and extracellular matrix organization (Figures 3(a)–3(c)). The results of KEGG (Kyoto Encyclopedia of Genes and Genomes) analysis suggested that these genes were enriched in tumor necrosis factor (TNF), nuclear factor kappa-B (NF-*κ*B), and PI3K-AKT signaling pathways (Figure 3(d)). These data demonstrated that quercetin may function in PTC by these pathways.

### 2.4. Molecular Docking Analysis

**Figure 4:**
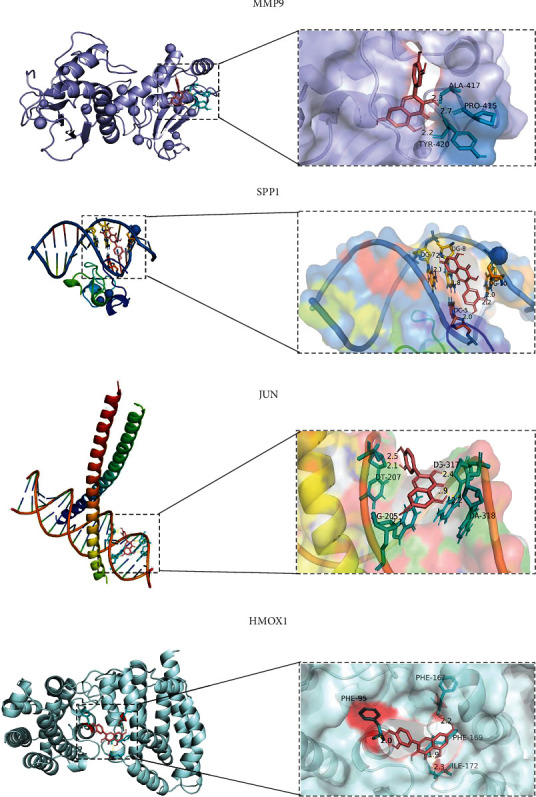
Top 4 proteins of quercetin designation and molecular docking analysis. Molecular docking data showed that the binding capacity of quercetin with PTC was significant in the key targets of MMP9, JUN, SPP1, and HMOX1. PTC: papillary thyroid carcinoma.

The results of molecular docking indicated the binding among quercetin and hub proteins, which may explain the mechanism of quercetin treatment in PTC in some ways. The molecular docking results showed that quercetin binds to central proteins, which may explain the mechanism of quercetin action on PTC to some extent. Quercetin inhibited the occurrence and progression of PTC by modulating MMP9, JUN, SPP1, and HMOX1 targets, which was in accordance with the results of NP screening. Molecular docking analysis validated the reliability of NP analysis. The results of quercetin docking with PTC target protein receptor sites are shown in Figure 4, and the top 10 molecular binding energies of quercetin docking with target proteins are shown in Table 2.

### 2.5. Quercetin Treatment Inhibited Cell Growth, Migration, and Invasion of PTC

**Figure 5:**
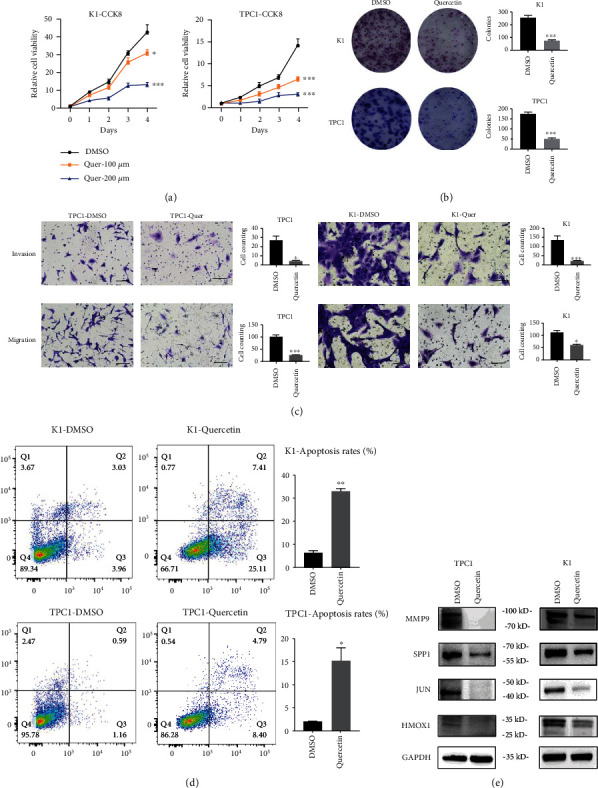
Quercetin blocked the proliferation of PTC cells in vitro. (a) CCK-8 assays and (b) colony formation assays showed that quercetin inhibited significantly suppressed cell proliferation in K1 and TPC1 cells. (c) Quercetin inhibited the migration and invasion of PTC cells in vivo. (d) Flow cytometry revealed increased apoptosis of K1 and TPC1 cells following quercetin treatment; *x*-axis represents FITC. (e) Western blotting analyses of MMP9, SPP1, JUN, and HMOX1 expression with or without quercetin treatment. The data are presented as the mean ± SD. All ^∗^*p* < 0.05, ^∗∗^*p* < 0.01, and ^∗∗∗^*p* < 0.001.

Colony formation and CCK-8 assays verified that quercetin treatment expressively suppressed cell proliferation of PTC cells with concentration dependence, as shown in Figures 5(a) and 5(b). Besides, quercetin treatment inhibited the migration and invasion of PTC cells (Figure 5(c)). Following quercetin treatment for 24 h, apoptosis rates increased (Figure 5(d)). These results indicate that quercetin blocked the self-renewal ability of PTC cells. Then, the expressions of the 4 genes MMP9, JUN, SPP1, and HMOX1 were measured by using western blotting. Compared with the control group, the 4 genes' expressions were downregulated by quercetin (Figure 5(e)).

## 3. Discussion

PTC is the most common pathological type of thyroid malignancy, accounting for approximately 80-90%. The role of microenvironment in thyroid carcinogenesis has been clarified [15]. The components of microenvironment (extracellular matrix components (ECM) and stromal cells) surround and support cancer cells, interacting with them through cell-cell and cell-extracellular matrix interactions [16]. These are consistent with the results we obtained; through GO analysis, we concluded that the role of quercetin in PTC is enriched in processes such as response to extracellular stimulus, oxidoreductase activity, and extracellular matrix organization.

The poor outcomes of cancers are partially caused by the interaction of the tumor with the surrounding immune microenvironment and the collective effect of these interactions during tumorigenesis and progression, promoting through immune tolerance and immune escape, leading to the body's incapacity to completely clear the tumor [17].

Extensive epidemiological evidence suggests that fruits and vegetables may prevent some human cancers and be related to a reduced risk of cancer-related mortality [18–22]. Anticancer and anti-inflammatory activities are a range of the main quercetin mechanisms of action [23–25]. Previous experiments have shown that quercetin prevents the growth of multifarious tumors, including ovarian, breast, colon, and gastric tumors, by suppressing the cell cycle and inducing apoptosis; moreover, the mechanism cell signaling pathways might involve PI3K and MAPK pathways [26]. Quercetin has been reported to increase expression of differentiation marker NIS in dedifferentiated/anaplastic thyroid cancer cells [27]. A study found that combined utilization of quercetin and sorafenib induced inhibition of cell growth and contributed to reduce PTC cell motility, with a significant increase of mRNA levels of E-cadherin expression and decrease of N-cadherin [28]. Another research found that quercetin suppressed the expression of Hsp90 and inhibited the growth of PTC cells, but the molecular mechanism of antiproliferation and apoptosis of Hsp90 was unclear [29].

Our results suggested that 23 target genes were regulated by quercetin in PTC cells. MMP9, JUN, SPP1, and HMOX1, with the top 4 degrees through PPI network analysis, were considered as the key genes. Matrix metalloproteinase 9 (MMP9), a key regulator of the extracellular matrix, has been regarded as an attractive therapeutic target, which participates in the degradation of many extracellular matrix proteins and also participates in inflammatory and oncological disorders [30]. MMP9 is critical for the remodeling of extracellular matrix components of the basement membrane such as type IV collagen and laminin [24]. Moreover, MMP9 regulates other cellular processes, and its expression and secretion are upregulated in pathological states such as cancer and chronic inflammation [31–35]. A study showed that MMP9 was overexpressed in PTC tissues and that targeted inhibition of MMP9 reduced migration and invasion of PTC cells [36]. Our results also confirm that MMP9 expression was downregulated in quercetin-treated PTC cells and that cell migration and invasion reduced as well.

JUN, a key component of dimeric transcription factor activator protein-1 (AP-1), plays a role in the transcriptional response to extracellular signaling [37]. A previous study showed that AP-1 expression in papillary thyroid carcinoma cancer tissue is positively correlated with a high incidence of disease progression, a high tumor recurrence rate, and unfavorable disease-free survival [38]. In our experimental group, JUN expression was downregulated in PTC cells after quercetin treatment. JUN might be used as a therapeutic molecular target to benefit a cure for PTC patients [39].

Secretory phosphoprotein 1 (SPP1), also known as bone bridge protein (OPN), is an integrin-binding glycophosphoprotein and has been shown to be overexpressed in many cancers such as hepatocellular carcinoma, lung cancer, prostate cancer, and breast cancer [40–42]. A study reported that SPP1 mRNA overexpressed in PTC samples [43] and the intensity of SPP1 staining was corresponding to the tumor size and lateral cervical lymph node metastasis (LNM) in human PTC [44]. Moreover, the level of SPP1 produced by macrophages has been shown to be related to the development of psammoma bodies (PBs) in PTC [45], which may be linked with the development of thyroid cancer. Our experiments showed that quercetin also regulates the expression of SPP1 in PTC.

In cancer cells, heme oxygenase-1 (HMOX1/HO-1) may improve cancer aggressiveness and resistance to therapy, resulting in poor prognosis [46]. Previous studies showed that HMOX1 was associated with advanced tumor stage in thyroid cancer [47].

According to the GO enrichment analysis, we found that biological process was focused on regulation of cell motility, cell adhesion mediated by integrin and response to extracellular stimulus including radiation, reactive oxygen species. Cellular component is enriched in extracellular matrix and membrane structure. As previously mentioned, MMP9 is a key regulator of the extracellular matrix and JUN plays a role in the transcriptional response to extracellular signaling; SPP1 is an integrin-binding glycophosphoprotein and HMOX1 overexpressed in cells such as macrophages supporting the immediate surroundings of the tumor [48], and the antioxidant role of HMOX1 in malignancies is well known [49].

Tumor-related pathways, such as PI3K-Akt, TNF, and NF-*κ*B signaling pathways, may be involved in quercetin treating PTC through KEGG enrichment analysis. Gene alterations caused by partners in the PI3K-AKT signaling pathway are closely associated with thyroid cancer progression [50]. NF-*κ*B is a key regulator of thyroid cancer angiogenesis [51]. For many years, it has been thought that the NF-*κ*B pathway in thyroid cancer cells plays a protumor role [52].

Our study suggested that quercetin may suppress PTC through the above mechanisms, which are closely related to key target proteins. The tumor signaling pathways and biological processes were described in the study. Further experimental and clinical validation of our findings is needed.

## 4. Methods

### 4.1. Screening Differentially Expressed Genes (DEGs) in PTC

From the Gene Expression Omnibus online database (https://www.ncbi.nlm.nih.gov/geo/query/acc.cgi?acc=GSE33630), we obtained the dataset (GSE33630) of gene expression in normal thyroid tissue and PTC. The results were assessed by utilizing the limma and pheatmap packages in R software.

### 4.2. Potential Targets of Quercetin

Quercetin chemical structure was acquired from PubChem (https://pubchem.ncbi.nlm.nih.gov/). Target proteins corresponding chemical small molecules were predicted by PharmMapper (http://www.lilab-ecust.cn/pharmmapper/).

### 4.3. Protein–Protein Interaction (PPI) Network Construction

The PPI network was constructed by utilizing the STRING 11.0 database (https://www.string-db.org/), and the mechanisms of these target protein interactions were visualized by Cytoscape 3.8.2 software. We entered the candidate target proteins into STRING to construct a PPI network; the minimum interaction score > 0.4. In the network, edge represented the PPI, while node represented target protein.

### 4.4. KEGG and GO Enrichment Analyses

KEGG pathway analysis and GO enrichment analysis were performed by utilizing R software. The topological analysis targets were imported; KEGG enrichment analysis and MF (molecular function), CC (cellular component), and BP (biological process) data were collected; weighting analyses were performed, and bubble diagrams were generated. The pathways concerned with PTC were determined by the clinical and pathological data; adjusted *p* values < 0.05.

### 4.5. Molecular Docking

The MOL2 format of quercetin's chemical structure (Figure 2(a)) was acquired from the Analysis Platform (TCMSP, Traditional Chinese Medicine Systems Pharmacology Database and Analysis Platform) database and then converting the file to PDB format using the PubChem database (https://www.pubchem.org/). The RCSB PDB database (http://www.rcsb.org/) was searched to obtain the protein crystal structure, and then, after dehydration and hydrogenation in AutoDock software, the structure was saved in PDB format. PyMOL software was used to analyze the quercetin-target proteins.

### 4.6. Cell Culture and Drug Treatment

TPC1 (PTC cell lines) was purchased from Guangzhou Cellcook Biotech Co. (Guangzhou, China); K1 was purchased from Shanghai Bangjing Industrial Co., LTD. (Shanghai, China). PTC cells were cultured in cell culture medium RPMI 1640 (Gibco) containing 10% fetal bovine serum and penicillin/streptomycin (100 units/mL, Gibco). The cells used for the experiments were from the 20–30 passage. Quercetin was obtained from Yuanye Biotechnology Co., Ltd. (Shanghai, China) and then dissolved in DMSO to concentrations of 100 mM and 200 mM.

### 4.7. Cell Viability and Colony Formation Assay

1000 cells per well were inoculated onto 96-well plates for cell counting and 6-well plates for colony formation. Cell viability was obtained by Cell Counting Kit-8 (CCK-8) assay. After culturing K1 and TPC1 cells with vehicle and quercetin (200 *μ*M) for 24 h, 1000 cells per well were reseeded in 6-well culture plates and cultured for 10 days and then visualized them by crystal violet staining.

### 4.8. Cell Invasion and Migration Assays

Invasion and migration assays were performed on 8 *μ*M pore polycarbonate membrane filters (Merck Millipore, USA) in 24-well plates. Transwell chambers were precoated with Matrigel (BD Biosciences, USA) for invasion assays, and Matrigel was not used for migration assays. The bottom of the culture chamber was placed with 600 *μ*L of culture medium containing 10% fetal bovine serum, while the upper chamber was full of 200 *μ*L of 1.2 × 10^4^ cells in 1640 culture medium incubated for 24 h. After fixation in 4% paraformaldehyde for 15 min, the chambers were stained with 0.2% crystal violet for 15 min.

### 4.9. Western Blot Analysis

Cells were lysed in RIPA buffer with 1% PMSF. Forty-five micrograms total protein was lysed by SDS–PAGE and then transferred to PVDF membranes (Millipore). The membranes containing protein molecules were immunoblotted overnight with primary antibodies. Primary antibodies applied to western blot analysis were anti-MMP9 (Cell Signaling Technology, 13667; 1 : 1000), anti-JUN (Cell Signaling Technology, 9165; 1 : 1000), anti-SPP1 (Santa Cruz Biotechnology, SC-21742; 1 : 500), anti-HMOX1 (Santa Cruz Biotechnology, sc-390991; 1 : 500), and anti-GAPDH (Cell Signaling Technology, 5174; 1 : 10000).

### 4.10. Flow Cytometric Analysis

Cells were treated with DMSO or quercetin for 24 h before flow cytometric apoptosis analysis. A million PTC cells were collected, then washed two times by cold PBS, and stained with APC-Annexin V/7-AAD or FITC-Annexin V/PI (Affymetrix eBioscience).

### 4.11. Statistical Analysis

Statistical analysis was performed by using SPSS 22.0 software (SPSS Inc., Armonk, NY, USA). The values of all counts were expressed as the mean standard deviation (SD) of 3 independent replicates. Comparative differences were tested using the Student *t*-test, with *p* < 0.05 and *p* < 0.01 indicating statistically significant differences.

## 5. Conclusions

In our study, quercetin systematically alters cell proliferation, invasion, and apoptosis of PTC cells by regulating the expression of several proteins such as MMP9, JUN, SPP1, and HMOX1. Through GO and KEGG enrichment analyses, we hypothesized that these protein molecules are mainly enriched in PI3K-Akt, NF-*κ*B, and TNF pathways. The study confirmed the anticancer effects and potential targets of quercetin in PTC.

## Figures and Tables

**Table 1:** Top 10 genes in the network.

Gene	Degree	Gene	Degree
MMP9	18	ICAM1	15
JUN	17	CXCL8	15
HMOX1	16	CCND1	14
SPP1	16	PPARG	14
MMP1	15	FOS	13

**Table 2:** Molecular docked binding energy of quercetin with the target proteins (kcal/mol).

	MMP9	JUN	SPP1	HMOX1
1	-6.05	-5.36	-6.47	-5.99
2	-5.28	-4.32	-6.46	-5.14
3	-5.16	-4.22	-5.54	-5.81
4	-5.12	-4.06	-5.39	-5.61
5	-4.64	-3.9	-5.95	-5.49
6	-4.33	-3.75	-5.5	-5.48
7	-4.32	-3.37	-5.22	-4.96
8	-4.18	-3.34	-5.15	-4.83
9	-4.17	-3.18	-5.11	-4.36
10	-4.05	-3.11	-4.02	-3.9

## Data Availability

The data that support the findings of this study are openly available in https://www.ncbi.nlm.nih.gov/geo/query/acc.cgiacc=GSE33630, https://pubchem.ncbi.nlm.nih.gov/, http://www.lilab-ecust.cn/pharmmapper/, https://www.pubchem.org/, http://www.rcsb.org/, and https://www.string-db.org/.
